# AST-120 Protects Cognitive and Emotional Impairment in Chronic Kidney Disease Induced by 5/6 Nephrectomy

**DOI:** 10.3390/brainsci14111043

**Published:** 2024-10-22

**Authors:** Yeon Hee Yu, Hyuna Im, Samel Park, Beomjong Song, Dae-Kyoon Park, Duk-Soo Kim, Hyo-Wook Gil

**Affiliations:** 1 Department of Anatomy, College of Medicine, Soonchunhyang University, Cheonan-si 31151, Republic of Korea; yyh0220@sch.ac.kr (Y.H.Y.); hyuna99012@sch.ac.kr (H.I.); beomjong.song@sch.ac.kr (B.S.); mdeornfl@sch.ac.kr (D.-K.P.); dskim@sch.ac.kr (D.-S.K.); 2 Department of Internal Medicine, Soonchunhyang University Cheonan Hospital, Cheonan-si 31151, Republic of Korea; samelpark17@schmc.ac.kr

**Keywords:** chronic kidney disease, AST-120, cognitive phenotypes, blood–brain barrier, indoxyl sulfate

## Abstract

Background: Uremic toxins resulting from chronic kidney disease (CKD) can cause cognitive and emotional disorders, as well as cardiovascular diseases. Indoxyl sulfate (IS) and p-cresol are notable uremic toxins found in patients with CKD. However, few studies have investigated whether reducing uremic toxins can alleviate cognitive and emotional disorders associated with CKD. Methods: We studied the effects of AST-120, which lowers IS levels, through behavioral tests, local field potentials, field excitatory postsynaptic potentials, and histological experiments in a 5/6 nephrectomy CKD model. Results: We confirmed AST-120’s effectiveness in CKD by measuring serum creatinine, blood urea nitrogen, and IS levels and performing renal tissue staining. Behavioral phenotypes indicated an alleviation of cognitive and anxiety disorders following AST-120 treatment in CKD-induced rats, which was further validated through local field potentials and field excitatory postsynaptic potential recordings. Double immunofluorescence staining for aquaporin-4 and glial fibrillary acidic protein in the hippocampus of CKD rats treated with AST-120 showed reduced coexpression. Conclusions: Our findings demonstrate the potential therapeutic effects of AST-120 in lowering IS levels and improving cognitive and emotional impairments associated with CKD.

## 1. Introduction

Chronic kidney disease (CKD) is a global health issue associated with complications, including cardiovascular disease, anemia, metabolic syndrome, and neurological complications [1]. Numerous clinical and laboratory studies have uncovered interactions between the kidneys and the brain, where traditional cardiovascular risk factors (such as diabetes, inflammation, hypertension, and dyslipidemia) and non-traditional risk factors related to kidney damage (such as uremic toxins) contribute to brain damage in CKD [2].

Single-nephron glomerular hyperfiltration is an early pathophysiologic manifestation of chronic kidney disease (CKD) and may lead to absolute glomerular hyperfiltration, characterized by a high glomerular filtration rate (GFR), or be associated with normal or low GFR due to nephron loss (relative glomerular hyperfiltration) [3]. Glomerular hyperfiltration during CKD progression is an important mechanism in humans. Our CKD animal model has confirmed an increase in blood urea nitrogen levels with the progression of CKD [4]. Additionally, cognitive impairment (CI) associated with CKD occurs not only in patients with end-stage renal disease (ESRD) but also in those with early-stage CKD, with the prevalence of CI in CKD estimated to be between 30% and 60% [5,6,7]. Our previous research also confirmed a significant reduction in hippocampal volume in ESRD patients who exhibited cognitive impairment (CI) [8]. A 5/6 nephrectomy in rats and mice is a good model for simulating renal failure following the loss of kidney function in humans. Moreover, our animal model demonstrated a decline in hippocampal-dependent memory with CKD progression, providing evidence of cognitive impairment regardless of age and a direct link between uremic toxins and cognitive dysfunction in CKD [8].

As renal function deteriorates in CKD, uremic toxins accumulate. Uremia is a biochemical and physiological dysfunction that increases with the progression of CKD [9]. Indoxyl sulfate (IS) and p-cresol are uremic toxins linked to the neurological complications of CKD [10,11,12,13]. Specifically, IS can activate pathways related to oxidative stress, nuclear factor erythroid 2-related factor 2, and mitogen-activated protein kinase signaling pathways [14]. Research indicates that IS triggers apoptosis in human astrocytes through oxidative stress, whereas long-term and high-dose exposures to IS inhibit the pathways associated with astrocytic cell death [15].

AST-120 (an oral spherical activated carbon) is an oral adsorbent consisting of porous carbon particles with diameters ranging from 0.2 to 0.4 mm. As a uremic toxin adsorbent, AST-120 absorbs low-molecular-weight compounds, including indole and p-cresol, precursors of IS and p-cresol sulfate (PCS), which result from intestinal amino acid metabolism [16]. Consequently, AST-120 reduces cardiovascular and chronic-kidney-disease-related complications by lowering serum IS levels and inhibiting the production of reactive oxygen species in endothelial cells, thus reducing oxidative stress [17]. However, little research has explored whether reducing IS levels alleviates cognitive and emotional impairment associated with CKD. Therefore, we investigated whether reducing IS with AST-120 could alleviate cognitive and emotional impairments in a 5/6 nephrectomy (5/6 Nx) CKD rat model.

## 2. Materials and Methods

### 2.1. Experimental Animals

All experiments were conducted using Sprague Dawley rats (8 weeks old, *n* = 35) sourced from the Experimental Animal Center of Soonchunhyang University (Cheonan, Republic of Korea). The rats had free access to water under controlled conditions of temperature, humidity, and lighting (light/dark cycle 12:12, and 22 ± 2 °C, 55 ± 5%). All animal protocols were approved by the Administrative Panel on Laboratory Animal Care at Soonchunhyang University (permit No. SCH22-0001, approval date: 15 February 2022). All possible efforts were made to reduce suffering and minimize the number of animals used.

### 2.2. 5/6 Nephrectomy CKD Rat Model

CKD was induced in rats by 5/6 nephrectomy (5/6 Nx, *n* = 20), as previously described [4]. Initially, rats were anesthetized with 2.5% isoflurane in a mixture of 33% oxygen and 67% nitrous oxide. The upper and lower poles of the left kidney were surgically removed, with sterile gauze applied to the exposed areas for 1–2 min to achieve hemostasis. Subsequently, a right-side nephrectomy was performed, resulting in a 5/6 reduction of renal mass.

### 2.3. Experimental Groups

The 5/6 Nx rats were randomly divided into two groups (*n* = 10 each): the first group received a normal diet (CKD) and the second received a normal diet supplemented with 8% AST-120 (Renamezin; Daewon Pharmaceutical Co., Ltd., Seoul, Republic of Korea) (CKD + AST-120). The concentration of AST-120 was chosen based on the findings of previous studies [18]. To compare the AST-120 dosage used in the CKD experiments with the human dosage, we calculated the human equivalent dose (HED) using the body surface area-based conversion method and the equation below [19].

HED (mg/kg) = animal dose (mg/kg) × (animal Km/human Km)

By applying the above equation, the human equivalent dose (HED) of AST-120 used in the experiment is approximately 2.04 g/kg per day. Based on a clinical dose of 6 g per day for a 60 kg adult, the 8% AST-120 used in the experiment falls within the permissible range. For comparison, age-matched rats on a standard diet (Control, *n* = 15) were used. Over 10 weeks, we monitored their body weight and food intake weekly. Following the guidelines set by the Institutional Animal Care and Use Committee of Soonchunhyang University, we used 3 to 5 animals per group for histological and electrophysiological experiments. In contrast, for behavioral studies, we typically used more than 5 animals per group to ensure sufficient power for non-parametric statistical analysis.

### 2.4. Serum Biochemical Assays

At 10 weeks post-surgery, blood samples were collected via cardiac puncture, and all serum biochemical assays were performed using commercially available kits. Blood urea nitrogen (BUN) was determined using a specific quantitative colorimetric assay (Quantichrome Urea Assay Kit) from BioAssay Systems (Hayward, CA, USA), following the manufacturer’s protocol. Serum creatinine levels were measured via another quantitative colorimetric assay (Creatinine Reagent Set) developed by POINTE SCIENTIFIC (Canton, MI, USA), in accordance with the manufacturer’s instructions. Serum total IS levels were assessed using high-performance liquid chromatography–fluorescence detection (HPLC-FLD, Agilent 1100 series; Agilent Technologies, Santa Clara, CA, USA) at an independent central laboratory (Seoul Clinical Laboratories, Seoul, Republic of Korea).

### 2.5. Behavioral Tasks

All rats from the three groups underwent behavioral assessments. These tests were monitored and analyzed using PC-based video behavior analysis and automated tracking software, Noldus EthoVision XT 14 (Noldus, Leesburg, VA, USA), and were conducted as outlined in previous research methods [4,8].

#### 2.5.1. Open-Field Test

The open-field test measuring anxiety was conducted as previously described [4]. Rats were placed in the central square (60 cm × 60 cm) of an open-field apparatus (60 cm × 60 cm × 40 cm) under diffuse lighting and allowed to explore freely for 30 min. Locomotor activity was evaluated based on the total distance moved, while anxiety levels were measured by the time spent in the center sector.

#### 2.5.2. Light–Dark Transition Test

The light–dark transition test involved a cage divided into two compartments, with a small opening allowing movement between them. One compartment was darkened (30 cm × 20 cm × 30 cm), while the other was brightly illuminated (30 cm × 30 cm × 30 cm). Rats were placed in the illuminated compartment and allowed to move freely for 5 min. The total time spent in the white compartment was used as an indicator of anti-anxiety behavior.

#### 2.5.3. Elevated Plus Maze Test

In the elevated plus maze test, rats were placed on an apparatus consisting of four arms elevated 60 cm above the floor, with two open arms (50 cm × 10 cm each) and two closed arms (50 cm × 10 cm each) featuring 50 cm high walls. The number of entries into the open arms and the duration spent there were measured over 5 min.

#### 2.5.4. Novel Objective Recognition Test

The novel object recognition test utilized a black acrylic box (60 cm × 60 cm × 40 cm) in the open-field test, and the animals were allowed to habituate to the box a day before the test for 30 min. On the habituation day, rats from all groups were reintroduced to the same chamber containing two identical objects. They were allowed to freely explore these objects for a total of 10 min and were then returned to their home cages. In the test phase, 24 h later, one of the objects was replaced by a novel object of a different shape and color. The rats were then placed back into the test chamber with the novel object and the familiar object for 10 min. In each phase, the animals were positioned at the center of the box, facing the wall opposite the two objects. Video reading and analysis were manually conducted. Object-exploring behaviors analyzed included accessing, sniffing, biting, and direct contact with the forelimb. The rats were assessed for their ability to recall the familiar object. Direct contact with an object, defined as object exploration (e.g., sniffing the object when <1 cm from it), was assessed. The discrimination index (DI) of the NOR test, defined as the ratio of time spent exploring the novel object to the time spent exploring both objects, was calculated as DI = (novel object exploration time − familiar object exploration time)/total exploration time.

#### 2.5.5. Y-Maze

The Y-maze consisted of 3 arms (40 cm × 5 cm × 20 cm; length, width, height) labeled A, B, and C, which converged at a 120° angle and were equidistant from each other. Each animal was placed at the end of one arm and allowed to explore freely for 8 min. The sequence of arm entries (e.g., ABC, BCA, CAB) and the total number of entries were manually recorded for each animal during the 8 min trial. Spontaneous alternation behavior, defined as consecutive entries into all three arms (e.g., ABC, CAB, or BCA but not ABA), was measured. The percentage (%) of spontaneous alternation behavior was calculated using the following formula: % alternation = [(number of alternations)/(total arm entries − 2)] × 100.

#### 2.5.6. Barnes Maze

The Barnes maze featured a circular platform, 122 cm in diameter, with 20 equally spaced holes around its perimeter, elevated 105 cm above the floor. Four distinct visual cues were strategically positioned on the walls of each quadrant at a height easily visible to the animals. An escape chamber located beneath one of these holes (escape hole) provided safety; bedding was added to this chamber to enable the animals to hide. An 80 Hz metronome and high-intensity lighting (300 lux at the center of the platform) were used to heighten anxiety and encourage the animals to find the escape hole. The animals engaged with the Barnes maze across three phases: habituation (1 day), training (2–4 days in extended training paradigms), and a probe (1 day). During the four-day adaptation phase, the rats were confined in a black cylinder at the platform’s center. After 10 s, upon removal of the cylinder, the animals began exploring the platform from the center, with their activity monitored by a video camera. The rats had 180 s to locate the escape hole. If a rat entered the escape chamber within 180 s, a cover was placed over the hole for 120 s to block the light and silence the electronic sound. If not, the animals were gently pulled and guided to the escape chamber hole to facilitate learning about the environment. To improve spatial memory for locating the escape hole, each animal underwent three tests at 15 min intervals during the training phase. On the fifth day, during the spatial acquisition phase (probe phase), the animals had 90 s to locate the escape hole, although the escape chamber had been removed. This probe was conducted once, as opposed to three times during the adaptation phase. The number of errors and the latency to reach the escape chamber were recorded and analyzed.

### 2.6. Local Field Potentials (LFPs)

Using previously published protocols, local field potentials (LFPs) were recorded in the hippocampus of rat brains [4,20]. After the behavioral task, each animal received an intraperitoneal injection of urethane (1.5 g/kg) and was placed in a stereotaxic frame. Holes were drilled through the skull for electrode insertion. In all animals, glass microelectrodes (microfilament capillary 1.2 outer diameter; 5–10 MΩ) filled with artificial cerebrospinal fluid (ACSF, in mM; NaCl 126, KCl 5, CaCl_2_ 2, MgCl_2_ 2, NaH_2_PO_4_ 1.25, NaHCO_3_ 26, D-glucose 10, pH 7.2) were employed. The coordinates (in mm) relative to bregma for accessing the dentate gyrus were 3.8 caudal, 2.5 lateral to bregma, and 2.9 in depth. Signals were recorded using a QP511 AC amplifier (0.1–3000 Hz bandpass, GRASS Technologies, West Warwick, RI, USA), digitized at 5 kHz, and analyzed with Clampfit 10.2 software (Axon Instruments, San Jose, CA, USA). LFP was monitored for 2 h. Single-channel electrical trace analysis was conducted using Clampfit 10.2 (Axon Instruments, San Jose, CA, USA). Changes in LFP’s normalized power were analyzed through amplitude spectrum analysis of normalized power estimated by event frequency. The root mean square (RMS) values were employed to calculate spectral power (mV^2^) at 1 Hz frequency bins for each electrode site. Spectral power values were averaged across all epochs within a single baseline, with the resultant power expressed as mV^2^/Hz. For each subject, a Fast Fourier Transform (FFT) of the epochs was calculated at a resolution of 0.61 Hz for all electrodes and then averaged. Non-overlapping Hamming windows were used to reduce spectral leakage. FFT power values were averaged across frequencies between 1 and 50 Hz to create 50 non-overlapping <1 Hz frequency bins, defining the frequency bands of interest as (24, 49, 50) δ (1–4 Hz), θ (4–7 Hz), and α (7–12 Hz).

### 2.7. In Vivo Field Excitatory Post-Synaptic Potential (fEPSP) Recording

fEPSPs were measured in the hippocampal CA1 region of rats, following previously described methods [8,21]. Animals were anesthetized intraperitoneally with urethane at a dose of 1.5 g/kg and placed in a stereotaxic frame. The rectal temperature was kept at 37 ± 0.3 °C during surgery with the help of a temperature controller (Harvard Instruments, Holliston, MA, USA). The scalp was incised and retracted. Holes were created in the skull to insert electrodes. The coordinates (in mm) relative to the bregma were as follows: for the recording electrode targeting the Schaffer collateral, 4.0 mm posterior to the bregma, 3.0 mm lateral to the midline, and 2.5 mm in depth. For the stimulating electrode targeting the stratum radiatum of CA1, 3.5 mm posterior to the bregma, 2.0 mm lateral to the midline, and 3.5 mm in depth. The optimal electrode depths were determined by maximizing the evoked response. The fEPSPs were adjusted to ~60% of the maximal response for testing. Stimulation was delivered using a BNC-2110 apparatus (National Instruments, Austin, TX, USA) and a Digital Stimulus Isolation unit (Getting Instruments, San Diego, CA, USA). The responses of pyramidal neurons to Schaffer collateral stimulation were recorded with a P55A.C. pre-amplifier (3–1000 Hz bandpass, Astro-Med Inc., West Warwick, RI, USA) and analyzed using WinLTP ver 2.01 software (WinLTP Ltd., Bristol, UK). Responses were elicited by single-pulse stimuli administered at 20 s intervals. A stable baseline was recorded for 30–60 min. Long-term potentiation (LTP) was induced using strong theta-patterned stimuli (sTPS), consisting of 4 trains of 10 bursts, each with 5 pulses at 400 Hz, with a 200 ms interval between bursts and a 15 s interval between trains. This induction is dependent on NMDA receptors. A robust LTP in the CA1 area was evoked by sTPS in vivo. To analyze changes in fEPSPs, the fEPSP slopes were averaged over 60 s intervals and expressed as percentages of the mean fEPSP slope measured during the 30 min baseline period, marked as 100%.

### 2.8. Tissue Processing and Double Immunofluorescent Staining

For immunostaining experiments, the animals were anesthetized with urethane (1.5 g/kg, i.p.) and perfused transcardially with phosphate-buffered saline (PBS) followed by 4% paraformaldehyde in 0.1 M PB. After extraction, the brains were post-fixed in the same solution for 4 h and then rinsed in PB containing 30% sucrose at 4 °C for 2 days. Subsequently, the tissues were sectioned using a microtome at 30 μm slices. To assess the therapeutic effects of AST-120 in a CKD animal model, we performed double immunofluorescence staining for GFAP and Aquaporin-4 (AQP-4), examining morphological changes in the blood–brain barrier (BBB). Brain tissues were incubated overnight at 4 °C in a solution containing mouse anti-GFAP IgG (Millipore, Burlington, MA, USA; diluted 1:500) and rabbit anti-AQP-4 IgG (Alomone Labs, Jerusalem, Israel; diluted 1:200). The sections were then washed with PBS and incubated in a solution containing Cy2- and Cy3-conjugated secondary antisera (Jackson Immuno Research Labs, West Grove, PA, USA; diluted 1:200) for 3 h at room temperature. Subsequently, the tissues were stained with DAPI (Invitrogen, Waltham, MA, USA; diluted 1:500) for 15 min at room temperature. The slices were washed with PBS and mounted on a slide with DPX (Sigma, St. Louis, MO, USA), and images were captured using a Fluoview FV10i microscope and FV10-ASW software (version 04.02, Olympus, Tokyo, Japan).

### 2.9. Quantification of Data and Statistical Analysis

Data quantification and statistical analysis were conducted as outlined in a previously described study, with some modifications [2]. The optical fractionator method was used to estimate the cell numbers in the molecular layer and CA1 regions. The sampling process is carried out by focusing through the tissue depth (with an optical dissector height of 30 μm in all cases for this study). All data were analyzed using GraphPad Prism 8.0.1 (GraphPad Software, San Diego, CA, USA) and are presented as the mean ± standard deviation. One-way analysis of variance (ANOVA), followed by Tukey’s multiple comparison test, was conducted, with *p*-values of <0.05, 0.01, and 0.001 considered statistically significant.

## 3. Results

### 3.1. General Characteristics and Effects of AST-120 on Renal Function

**Figure 1:**
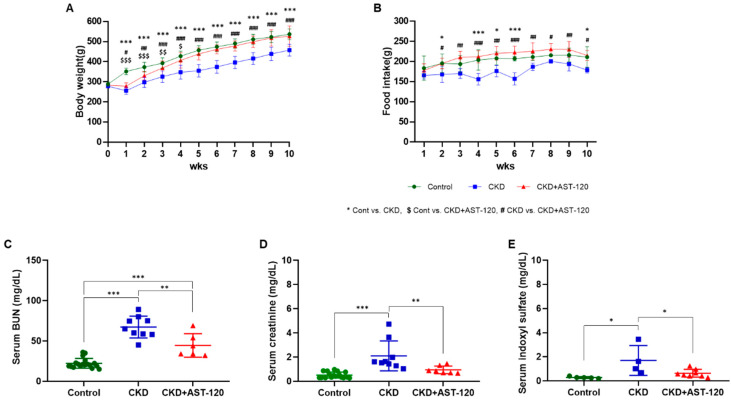
The effect of AST-120 treatment on body weight and blood analysis in the CKD group. Body weight and food intake in the CKD + AST-120 group were significantly restored to control levels (**A**,**B**). * *p* < 0.05 and *** *p* < 0.001, control group vs. CKD group; $ *p* < 0.05, $$ *p* < 0.01 and $$$ *p* < 0.001, control group vs. CKD + AST-120 group; # *p* < 0.05, ## *p* < 0.01 and ### *p* < 0.001, CKD group vs. CKD + AST-120 group. Serum BUN and creatinine levels were significantly decreased in the CKD + AST-120 group compared to the CKD group (**C**,**D**). Additionally, serum IS levels were significantly reduced in the CKD + AST-120 group compared to the CKD group (**E**). Data are presented as means ± standard errors of the mean (SEM). * *p* < 0.05, ** *p* < 0.01, *** *p* < 0.001, one-way analysis of variance (ANOVA) followed by Tukey’s post hoc multiple comparisons test. Body weight and food intake: *n* = 10/group.

To investigate the effects of IS reduction by AST-120 in the brain in CKD, we conducted behavioral, electrophysiological, and histological studies. Initially, the CKD group exhibited significantly reduced body weight and food intake compared to the control group. However, body weight and food intake in the AST-120-treated CKD group returned to control levels (Figure 1A,B). Serum BUN and creatinine levels in the CKD + AST-120 group (BUN, F_1,14_ = 5.954, *p* < 0.001; creatinine, F_1,15_ = 4.772, *p* < 0.01; Figure 1C,D) were lower than those in the CKD group (BUN, F_1,26_ = 15.16, *p* < 0.001; creatinine, F_1,25_ = 8, *p* < 0.001; Figure 1C,D). Additionally, the serum IS level was decreased in the CKD + AST-120 group (F_1,11_ = 3.964, *p* < 0.05; Figure 1E) compared to the CKD group (F_1,8_ = 4.827, *p* < 0.05; Figure 1E).

### 3.2. Effects of AST-120 on Renal Fibrosis

**Figure 2:**
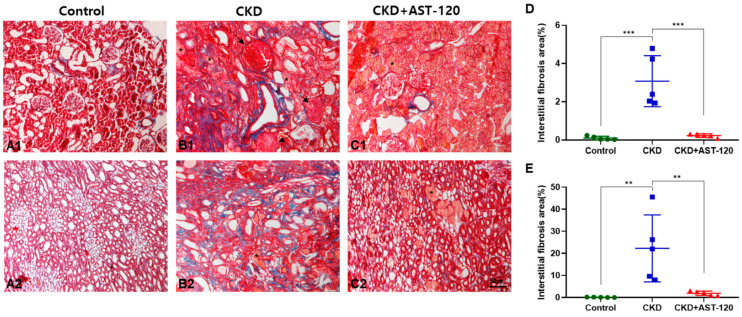
The effect of AST-120 treatment on renal fibrosis in CKD rats. Representative images of the cortex (**A1**–**C1**) and medulla (**A2**–**C2**) of the kidney in the control, CKD, and CKD + AST-120 groups. Masson’s trichrome staining revealed progressive interstitial fibrosis in the CKD group, which significantly decreased in the CKD + AST-120 group (**A**–**E**). Notably, the CKD group exhibited glomerulosclerosis (arrow) and injured tubules (*) (**B1**,**B2**). Glomerulosclerosis in the CKD + AST-120 group was significantly reduced compared to that in the CKD group (**C1**). However, injured tubules in the CKD + AST-120 group did not show recovery (*) (**C1**,**C2**). Data are presented as means ± SEM. ** *p* < 0.01, *** *p* < 0.001, one-way ANOVA followed by Tukey’s post hoc multiple comparisons test. *n* = 5/group.

We confirmed the effect of AST-120 on fibrosis using Masson’s trichrome staining of renal tissue (Figure 2). Progressive interstitial fibrosis was observed in the cortex and medulla of the CKD group compared to the control group (cortex, F_1,9_ = 8.601, *p* < 0.001; medulla, F_1,9_ = 5.651, *p* < 0.01; Figure 2(A1–B2),D,E). However, the CKD group treated with AST-120 showed a remarkable reduction in progressive interstitial fibrosis compared to the CKD group (cortex, F_1,9_ = 8.216, *p* < 0.001; medulla, F_1,9_ = 5.188, *p* < 0.01; Figure 2(B1–C2),D,E). In particular, increased glomerulosclerosis (arrow) in the CKD group was significantly alleviated by AST-120 treatment (Figure 2(B1,C1)), while injured tubules (*) did not recover with AST-120 treatment (Figure 2(B2,C2)).

### 3.3. Alleviation of Anxiogenic Phenotypes Through AST-120 Treatment After CKD

**Figure 3:**
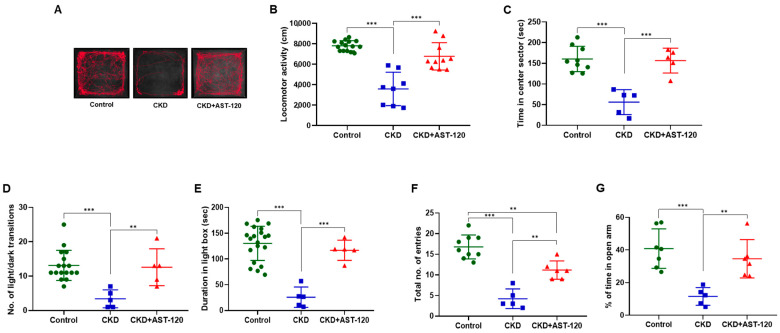
The impact of AST-120 treatment on anxiogenic phenotypes in the CKD group. Representative cumulative traces of navigational pathways for the control, CKD, and CKD + AST-120 groups during exploratory behavior in the open field (**A**). Locomotor activity and time spent in the central sector by the CKD + AST-120 group were higher compared to the CKD group (**B**,**C**). Light/dark transition counts and total duration in the light box were greater in the CKD + AST-120 group than in the CKD group (**D**,**E**). The total number of entries and the percentage of entries into the open arms of the elevated plus maze were markedly enhanced in the CKD + AST-120 group compared to the CKD group (**F**,**G**). Data are presented as means ± SEM. ** *p* < 0.01, and *** *p* < 0.001, one-way ANOVA followed by Tukey’s post hoc multiple comparisons test.

To determine if AST-120 treatment alleviates locomotor and anxiety disorders induced by CKD, we analyzed the levels of locomotor and anxiety-related behaviors in the CKD group and the AST-120-treated CKD group. (Figure 3). In the open-field assay, the CKD group displayed reduced locomotion compared to the control group (F_1,22_ = 12.08, *p* < 0.001; Figure 3A,B); however, locomotion levels in the CKD group treated with AST-120 were restored to those of the control group (vs. CKD rats, F_1,17_ = 8.426, *p* < 0.001; Figure 3A,B). Additionally, the CKD group treated with AST-120 (F_1,9_ = 7.399, *p* < 0.001; Figure 3C) spent more time in the central sector of the open field than the untreated CKD group (F_1,13_ = 8.718, *p* < 0.001; Figure 3C). In the light/dark box test, the CKD group showed fewer transitions from the dark to the light compartment (F_1,21_ = 6.237, *p* < 0.001; Figure 3D) and spent less time in the light chamber (F_1,24_ = 9.905, *p* < 0.001; Figure 3E) than the control group. However, the CKD group treated with AST-120 demonstrated increased transitions from the dark to the light compartment (F_1,9_ = 4.75, *p* < 0.01; Figure 3D) and more time spent in the light chamber (F_1,9_ = 6.825, *p* < 0.001; Figure 3E), akin to the control group. Similarly, in the elevated plus maze task, the CKD group made fewer total entries (closed arms plus open arms) (F_1,13_ = 12.25, *p* < 0.001; Figure 3F) and spent less time in the open arms (F_1,11_ = 6.686, *p* < 0.001; Figure 3G) compared to the control group, but the CKD group treated with AST-120 exhibited increases in both parameters compared to the untreated CKD group (total number of entries, F_1,10_ = 6.251, *p* < 0.01; time spent in the open arms, F_1,10_ = 5.088, *p* < 0.01; Figure 3F,G). Thus, our findings indicate that AST-120 treatment mitigates locomotor and anxiety-related behaviors induced in the group following CKD.

### 3.4. Alleviation of Cognitive Behavioral Deficits Following Effects of AST-120 After CKD

**Figure 4:**
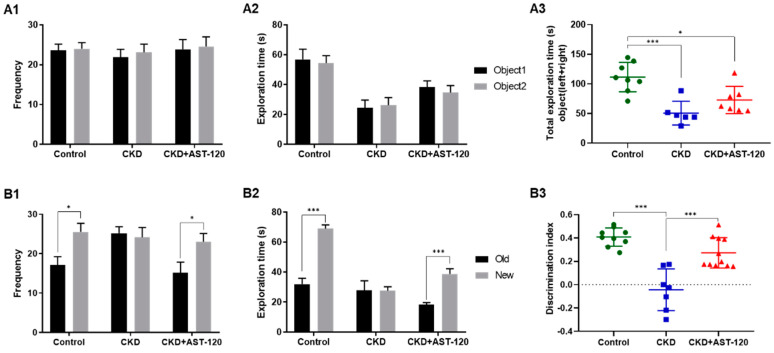
The effect of AST-120 treatment on novel object recognition memory in the CKD group. During the habituation period, the frequency and exploration time for the same objects were similar among the control, CKD, and CKD + AST-120 groups (**A1**,**A2**). The total object exploration times were significantly restored in the CKD + AST-120 group compared to the CKD group (**A3**). Additionally, the CKD + AST-120 group exhibited significantly increased frequency and time spent on novel objects, comparable to the control group (**B1**,**B2**). Specifically, the discrimination index for novel objects in the CKD + AST-120 group was notably higher compared to the CKD group (**B3**). Data are presented as means ± SEM. * *p* < 0.05 and *** *p* < 0.001, one-way ANOVA followed by Tukey’s post hoc multiple comparisons test.

**Figure 5:**
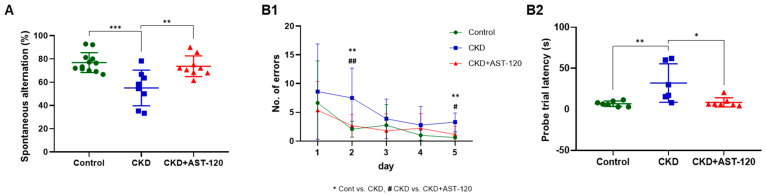
The effect of AST-120 treatment on spatial working and learning memory in the CKD group. In the CKD + AST-120 group, the average percentage of spontaneous movement was markedly reduced compared with the CKD group (**A**). The CKD + AST-120 group demonstrated fewer errors on the second and fifth days of testing (probe test) compared to the CKD group (**B1**). ** *p* < 0.01, control group vs. CKD group; # *p* < 0.05 and ## *p* < 0.01, CKD group vs. CKD + AST-120 group. Notably, in the CKD + AST-120 group, latency times during the probe trials to find the target hole were restored to control group levels (**B2**). Data are presented as means ± SEM. * *p* < 0.05, ** *p* < 0.01, and *** *p* < 0.001, one-way ANOVA followed by Tukey’s post hoc multiple comparisons test.

To assess the impact of AST-120 on cognitive deficits, cognitive-related behavioral analyses were conducted, including novel object recognition (NOR), Y-maze, and Barnes maze (Figure 4 and Figure 5). In the NOR test, for the exploration frequency and the time of control, the CKD and AST-120-treated CKD groups demonstrated similar exploration patterns between the same two objects (Figure 4(A1,A2)). The total object exploration times were significantly lower in the CKD group than in the control group (F_1,13_ = 6.917, *p* < 0.001; Figure 4(A3)); however, this decrease was mitigated in the CKD + AST-120 group (vs. control, F_1,14_ = 4.574, *p* < 0.05; Figure 4(A3)). The CKD group did not differ in frequency and time spent on a novel object (Figure 4(B1,B2)). Yet, the AST-120-treated CKD group showed a significant increase in frequency and exploration time searching for a novel object, comparable to the control group (frequency, *p* < 0.05; exploration time, *p* < 0.001; Figure 4(B1,B2)). The discrimination index was substantially reduced in the CKD group compared to the control group (F_1,15_ = 9.681, *p* < 0.001; Figure 4(B3)), but it approached control levels in the AST-120-treated CKD group (vs. CKD, F_1,17_ = 7.076, *p* < 0.001; Figure 4(B3)). In the spatial memory test, the CKD group exhibited fewer spontaneous alterations than the control group (F_1,19_ = 6.233, *p* < 0.001; Figure 5A). However, spontaneous alterations were increased in the AST-120-treated CKD group, similar to the control group (F_1,16_ = 5.01, *p* < 0.01; Figure 5A). The Barnes maze apparatus was utilized to assess spatial learning and memory in animal models (Figure 5(B1,B2)). Through repeated trials using the Barnes maze, the number of escape errors diminished across all rat groups (Figure 5(B1)). Notably, the untreated CKD group exhibited a higher number of errors in finding the escape hole on training day 2 and during the probe trial (training days 2, F_1,12_ = 4.966, *p* < 0.01; probe trial, F_1,12_ = 4.833, *p* < 0.01; Figure 5(B1)), but these errors were significantly reduced in the AST-120-treated CKD group (training days 2, F_1,12_ = 4.636, *p* < 0.01; probe trial, F_1,12_ = 4.367, *p* < 0.05; Figure 5(B1)). Furthermore, the increased target latency observed in the CKD group (F_1,12_ = 4.821, *p* < 0.01, Figure 5(B2)) during the probe test day was significantly reduced to the level of the control group in the CKD group treated with AST-120 (F_1,12_ = 4.509, *p* < 0.05; Figure 5(B2)).

### 3.5. Representative Profile of LFP Following Effects of AST-120

**Figure 6:**
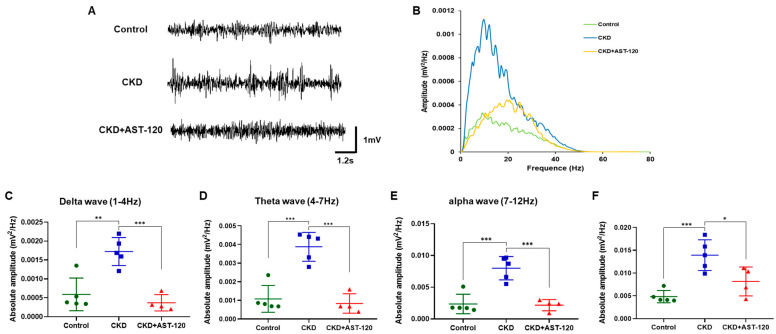
The effect of AST-120 treatment on theta-frequency oscillations in the CKD group. Representative raw traces of LFP signals in the CA1 region of the hippocampus (**A**). Power spectral analysis of the CKD + AST-120 group showed significant reductions in power at lower frequencies compared to the CKD group (**B**). Hippocampal delta, theta, and alpha oscillations were significantly decreased in the CKD + AST-120 group, approximating levels seen in the control group (**C**–**E**). The average normalized power of the CKD + AST-120 group was notably lower compared to the CKD group (**F**). Data are presented as means ± SEM. * *p* < 0.05, ** *p* < 0.01 and *** *p* < 0.001, one-way ANOVA followed by Tukey’s post hoc multiple comparisons test. Control: *n* = 5; CKD: *n* = 5; CKD + AST-120: *n* = 4.

To determine if AST-120 treatment could restore altered theta-frequency oscillations, we conducted LFP recordings in the hippocampal CA1 region of the control group, CKD group, and AST-120-treated CKD group under urethane anesthesia (Figure 6). In the CKD group, we observed epileptic discharges as irregular sharp waves and multiple spikes, consistent with prior reports (Figure 6A) [4]. However, AST-120 treatment alleviated these epileptic discharges in the CKD group (Figure 6A). Furthermore, the power spectral density of LFP increased significantly in the CKD group but was reduced in the AST-120-treated CKD group (Figure 6B). Power spectral analysis demonstrated that the absolute power of delta (F_1,9_ = 7.015, *p* < 0.01; Figure 6C), theta (F_1,9_ = 9.017, *p* < 0.001; Figure 6D), and alpha (F_1,9_ = 8.3, *p* < 0.001; Figure 6E) oscillations significantly increased in the CKD group, while it markedly decreased in the AST-120-treated CKD group compared to the untreated CKD group (delta, F_1,8_ = 7.908, *p* < 0.001; theta, F_1,8_ = 9.248, *p* < 0.001; alpha, F_1,8_ = 8.086, *p* < 0.001; Figure 6C–E). The normalized LFP power in the CKD group was significantly higher compared to the control group (F_1,9_ = 8.665, *p* < 0.001; Figure 6F). However, normalized LFP power in the CKD group treated with AST-120 decreased to resemble that of the control group (vs. CKD, F_1,8_ = 6.498, *p* < 0.01; Figure 6F). Collectively, these findings suggest that AST-120 treatment mitigates alterations in theta-frequency oscillations, a neural oscillatory marker for anxiety disorder following CKD.

### 3.6. Alterations of fEPSP in Hippocampal Neurons Following Effects of AST-120 After CKD

**Figure 7:**
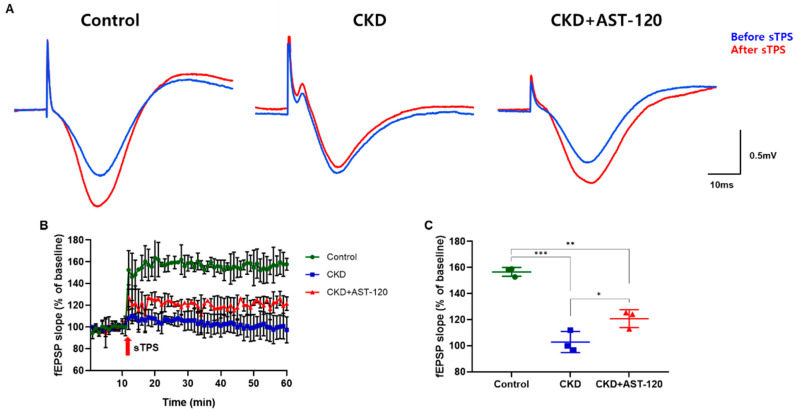
The effect of AST-120 treatment on fEPSP in the CKD group. Representative traces for fEPSP in the hippocampal CA1 region before and after sTPS in the control, CKD, and CKD + AST-120 groups (**A**). The amplitude of evoked fEPSP (**B**) and the slope changes (**C**) following LTP in the CKD + AST-120 group was significantly increased compared with the CKD group. The arrows in panel B indicate the time point of the sTPS application for LTP. Data are presented as means ± SEM. * *p* < 0.05, ** *p* < 0.01, and *** *p* < 0.001, one-way ANOVA followed by Tukey’s post hoc multiple comparisons test, *n* = 3/group.

We investigated the effect of AST-120 on learning and memory in CKD by examining the synaptic plasticity induced by N-methyl-D-aspartate (NMDA) receptor-mediated long-term potentiation (LTP) (Figure 7). sTPS stimulation consistently induced LTP in the CA1 region (Figure 7A). In the CKD group, both the amplitude and slope of the evoked fEPSP following sTPS application were markedly diminished compared to the control group (F_1,5_ = 14.55, *p* < 0.001; Figure 7B,C). However, in the CKD group treated with AST-120, the amplitude and slope of the evoked fEPSP markedly increased compared to the untreated CKD group (F_1,5_ = 4.847, *p* < 0.05; Figure 7B,C).

### 3.7. Reduction in Gliosis Due to Effects of AST-120 in CKD

**Figure 8:**
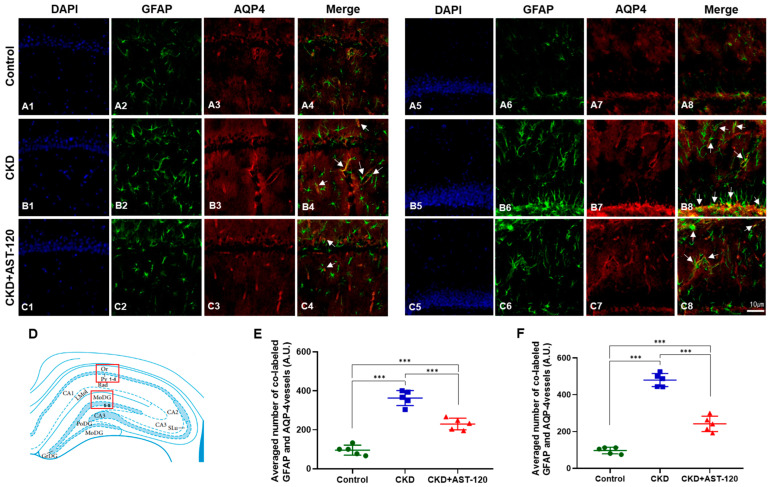
The effect of AST-120 treatment on cerebral edema in the hippocampus in the CKD group. The double labeling of GFAP and AQP-4 in the hippocampal CA1 (**A1**–**A4**) and DG (**A5**–**A8**) regions of the control group revealed significantly reduced GFAP immunoreactivity in the hippocampus of the CKD + AST-120 group in the CA1 (**B1**–**C2**) and DG (**B5**–**C6**) regions compared to the CKD group. Furthermore, double labeling of GFAP and AQP-4 was markedly decreased in the hippocampal CA1 (arrows, **B3**–**C4**) and DG (arrows, **B7**–**C8**) regions of the CKD + AST-120 group relative to the CKD group. GFAP (green); AQP-4 (red); merged images (yellow); scale bar = 10 μm. A diagram of the hippocampal CA1 and DG (**D**) regions. There was a significant difference in quantification between the CKD + AST-120 group and the CKD group (**E**,**F**). A.U.—arbitrary unit. Data are presented as means ± SEM. *** *p* < 0.001, one-way ANOVA followed by Tukey’s post hoc multiple comparisons test, *n* = 5/group.

Double-immunofluorescence for GFAP and AQP4 was performed to assess functional changes in astroglia and the blood–brain barrier following AST-120 treatment (Figure 8). Markedly elevated GFAP-positive immunoreactivity in astroglia was observed in the hippocampal CA1 region and the molecular layer of the DG in the CKD group compared to the control group (Figure 8(A1–B2,A5–B6)). In contrast, the CKD group treated with AST-120 showed a marked reduction in GFAP-positive astrocytes compared to the untreated CKD group (Figure 8(C1,C2,C5,C6)). In the CKD group, AQP4-positive immunoreactivity in astrocytes and blood vessels was markedly elevated in the CA1 and molecular layer of the DG region compared with the control group (CA1, F_1,9_ = 18.77, *p* < 0.001; DG, F_1,9_ = 25.57, *p* < 0.001; Figure 8(A3–B4,A7–B8),E,F). However, the CKD group treated with AST-120 exhibited a significant decrease in the double labeling of AQP4 and GFAP in blood vessels compared to the untreated CKD group (CA1, F_1,9_ = 9.345, *p* < 0.001; DG, F_1,9_ = 15.89, *p* < 0.001, arrows; Figure 8(C3,C4,C7,C8),E,F).

## 4. Discussion

Our study demonstrated that AST-120 significantly attenuated CKD-induced cognitive and emotional impairments and reduced hippocampal injury and BBB disruption by lowering serum IS in 5/6 nephrectomy rats.

The pathogenesis of CKD-induced cognitive impairment involves vascular injury and uremic toxins. Although common findings in CKD include vascular injury, such as atherosclerosis and endothelial dysfunction, recent studies have shown that uremic toxins also contribute to cognitive impairment in these patients [22]. Our previous research confirmed that uremia induced by CKD leads to hippocampal damage, which results in cognitive and emotional functional impairments [8].

According to previous studies, mice injected with PCS after CKD induction exhibited potential impairments in emotional and spatial learning due to the accumulation of PCS, which could be alleviated by AST-120 [13]. In the CKD model, PCS injection increases NOX4 and decreases Nrf2/HO-1, leading to oxidative stress and an elevation in microglial-related CD68 protein, indicating a potential role in neuroinflammation [13].

However, clinically, serum IS levels, but not P-cresol, are negatively correlated with cognitive function in CKD patients [11,23]. Li et al. demonstrated that AST-120 effectively reduces serum and hippocampal IS levels and reverses cognitive impairment in CKD mice [24]. In our current study, treatment with AST-120 in CKD reduced serum IS levels. Furthermore, AST-120 treatment was found to alleviate both cognitive impairment and emotional disturbances. Additionally, we demonstrated through local LFP that AST-120 mitigates the increase in theta and alpha rhythms induced by CKD, thereby restoring processing abilities related to anxiety and cognition [25,26]. Particularly, AST-120 has been shown to be effective in cognitive functions such as learning and memory by alleviating impairment in activity-dependent synaptic plasticity in the hippocampus of CKD rats. This suggests that compared to other uremic toxins, IS is a significant neurotoxin associated with cognitive functions, and reducing its level could lead to improvements.

The inflammatory response in CKD patients is associated with disease progression and closely correlates with kidney function and IS levels [22]. Reactive microglia release various cytokines and chemokines, such as IL-1, IL-2, IL-6, tumor necrosis factor alpha (TNF-α), and interferon gamma (IFN-γ), which stimulate astrocyte activity and promote a neuroinflammatory response [27]. Prior research indicates that abnormal cytokine production is linked to cognitive impairment in hemodialysis patients [28]. Additionally, severe or prolonged systemic inflammation activates microglia, exacerbating cognitive issues, including synaptic loss, dendritic spine changes, neuronal cell death, neurogenesis impairment, memory deficits, and hypothalamic dysfunction [29]. Recent studies show that IS is a significant uremic toxin inducing NLRP3 inflammasome-mediated cognitive impairment in microglia and astrocytic inflammation [24]. In animals with renal dysfunction, IS sulfate exacerbated cognitive impairment by disrupting the BBB [30]. Our previous research confirmed increased BBB permeability and cognitive impairment resulting from CKD [8]. AQP-4 and GFAP, as astrocyte markers, have been associated with various physiological and pathological conditions in the central nervous system, including BBB breakdown [31]. Astrocytic ion channels play a central role in central nervous system (CNS) function and homeostasis by transporting ions, absorbing neurotransmitters, and generating neurotrophic factors [32]. In this study, we observed that AST-120 treatment attenuated increased astrocyte co-expression with AQP-4 in the hippocampus of CKD rats. Previous studies have shown that AST-120 reduces blood uremic toxins and improves the function of the BBB [30,33]. Our findings support that AST-120 mitigated the BBB breakdown induced by increased IS levels in CKD. In summary, these results suggest that the reduction in serum IS levels due to AST-120 decreases BBB permeability, ultimately alleviating cognitive impairment. However, further research is needed to clarify whether the reduction in cognitive impairment due to AST-120 is a direct result of changes in hippocampal aquaporin-4 and glial fibrillary acidic protein levels or a secondary effect of decreased uremic toxin levels. Additionally, these results suggest that while it is difficult to directly determine whether AST-120 crosses the BBB or lowers blood concentrations, it is undeniable that AST-120 improves the impaired BBB function caused by CKD. However, future studies to confirm the passage of AST-120 across the BBB will undoubtedly be an important mechanism for explaining the potential of AST-120.

Our study has some limitations. First, we did not assess vascular conditions. However, since CKD was induced in our experimental rats during their young adult stage, we can rule out cognitive dysfunction caused by age-related vascular damage. Second, we did not measure IS levels in brain regions. However, in a previous study, neurobehavioral deterioration associated with increasing IS levels in brain regions was confirmed [30]. Additionally, in a CKD model, we found that AST-120 reduced blood IS levels and restored the BBB [33]. By combining the results of previous studies with our findings, we suggest that AST-120 effectively reduces IS levels in the hippocampus.

## 5. Conclusions

This study establishes a critical link between elevated IS levels in CKD and cognitive dysfunction, emphasizing the potential therapeutic impact of AST-120 in ameliorating cognitive and emotional deficits. Clinically, AST-120 is primarily used to slow the progression of kidney dysfunction in CKD. However, if clinical studies confirm that AST-120 delays cognitive decline in CKD, it could become a key therapeutic target for improving cognitive and emotional function in metabolic encephalopathy or CKD. By reducing IS levels, AST-120 emerges as a promising intervention to enhance cognitive function in CKD, suggesting avenues for future therapeutic strategies targeting uremic toxins in neurocognitive disorders associated with renal dysfunction.

## Data Availability

The original contributions presented in the study are included in the article; further inquiries can be directed to the corresponding author.
